# Accessibility and Usability of State Health Department COVID-19 Vaccine Websites: A Qualitative Study

**DOI:** 10.1001/jamanetworkopen.2021.14861

**Published:** 2021-05-26

**Authors:** Jessica L. Howe, Chelsea R. Young, Codrin A. Parau, J. Gregory Trafton, Raj M. Ratwani

**Affiliations:** 1 Medstar Health National Center for Human Factors in Healthcare, Washington, DC; 2 Department of Psychology, George Mason University, Fairfax, Virginia; 3 Naval Research Laboratory, Washington, DC; 4 Georgetown University School of Medicine, Washington, DC

## Abstract

This qualitative study analyzes department of health websites for all 50 US states for accessibility and usability in obtaining COVID-19 vaccine eligibility information and appointments.

## Introduction

The US Centers for Disease Control and Prevention allocates vaccines to individual states for distribution.^1^ Individuals seeking COVID-19 vaccine eligibility information and appointments must locate the resources provided in their state, making state department of health websites a primary source of information. Effective and equitable vaccine distribution depends, in part, on the accessibility and usability of these websites. Different levels of technology experience, reading abilities, and language preferences should not prevent individuals from obtaining needed vaccine information. Website usability should support determining vaccine eligibility, provide an indicator of when information was last updated, and provide web-based appointment scheduling and/or a wait-list and follow-up process. The vaccine rollout has resulted in frustration and inequities among underserved racial/ethnic groups.^2,3^ We analyzed each state’s department of health website for accessibility and usability challenges.

## Methods

This qualitative study was not submitted for institutional review board approval and informed consent was not needed or sought because it does not involve human participants or their data. This study follows the Standards for Reporting Qualitative Research (SRQR) reporting guideline.

Each state’s department of health website with vaccine eligibility and availability information, or designated website alternative, was reviewed February 5 to 8, 2021. Accessibility and usability were determined for all 50 states in the context of a person seeking to determine vaccine eligibility, schedule a web-based appointment, or be added to a wait-list for follow-up.

Accessibility measures included website viewability by computer and smartphone, availability of non-English language options, web accessibility score (0%-100%), with higher scores indicating greater adherence to rules for perceivability and understandability by diverse populations,^3^ and Flesch-Kincaid readability score for eligibility information, with lower scores indicating lower grade level required for comprehension.^4,5^ Usability evaluation was based on adherence to design heuristics.^6^ We analyzed whether websites provided an indicator of when the information was last updated (visibility of system status heuristic), step-by-step form or similar process to help users determine their eligibility for a vaccine to prevent ineligible users from seeking appointments (error prevention heuristic), and appointment scheduler or wait-list or follow-up option (efficiency of use heuristic). Data were collected using Excel 365 (Microsoft).

## Results

**Figure 1.**
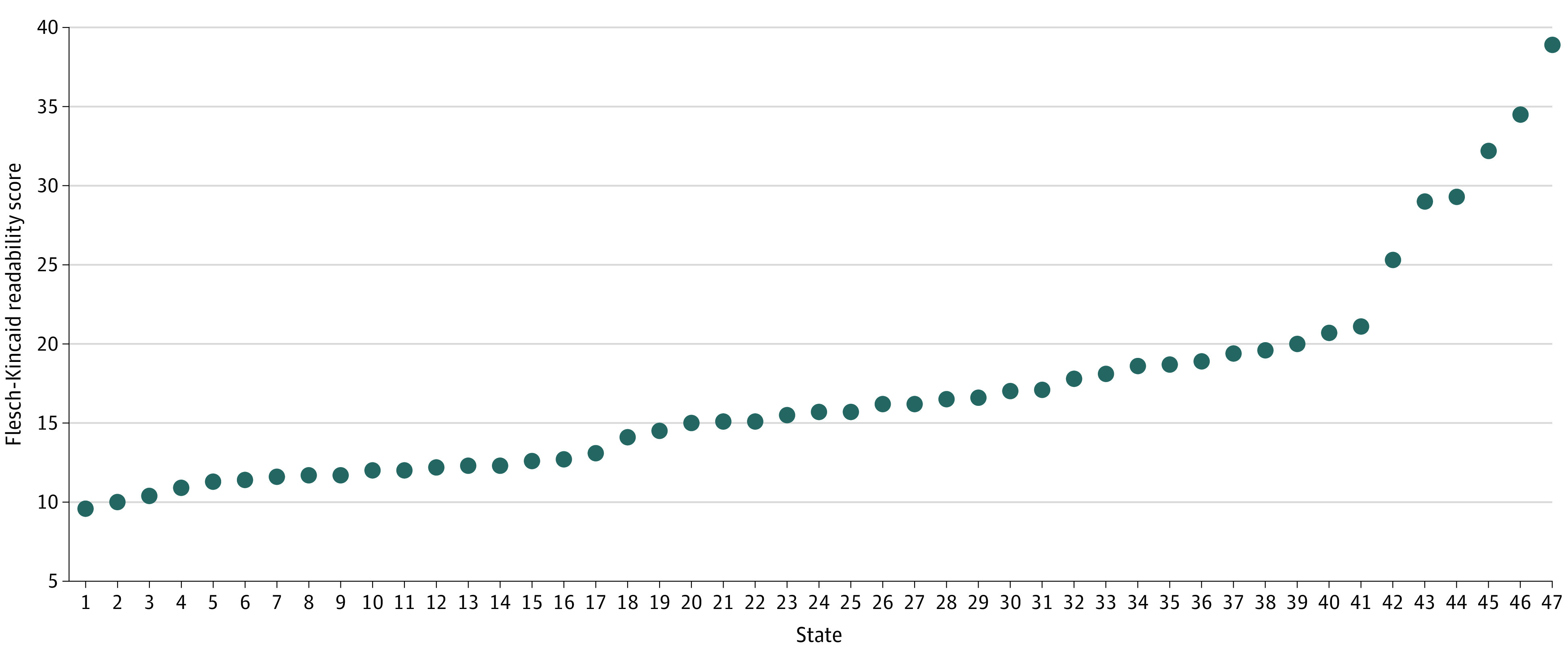
Readability Score (Flesch-Kincaid Grade Level) for 47 State Websites Providing Eligibility Information

All websites were viewable by computer. Seven states (14%) did not enable complete smartphone viewability, and 9 states (18%) were English only. The mean (SD) web accessibility score was 82% (4%). Forty-seven states (94%) presented eligibility information with median (range) readability scores of 15.6 (9.5-38.9), and most (39 of 47 [83%]) did so at a post–high-school reading level (Figure 1).

**Figure 2.**
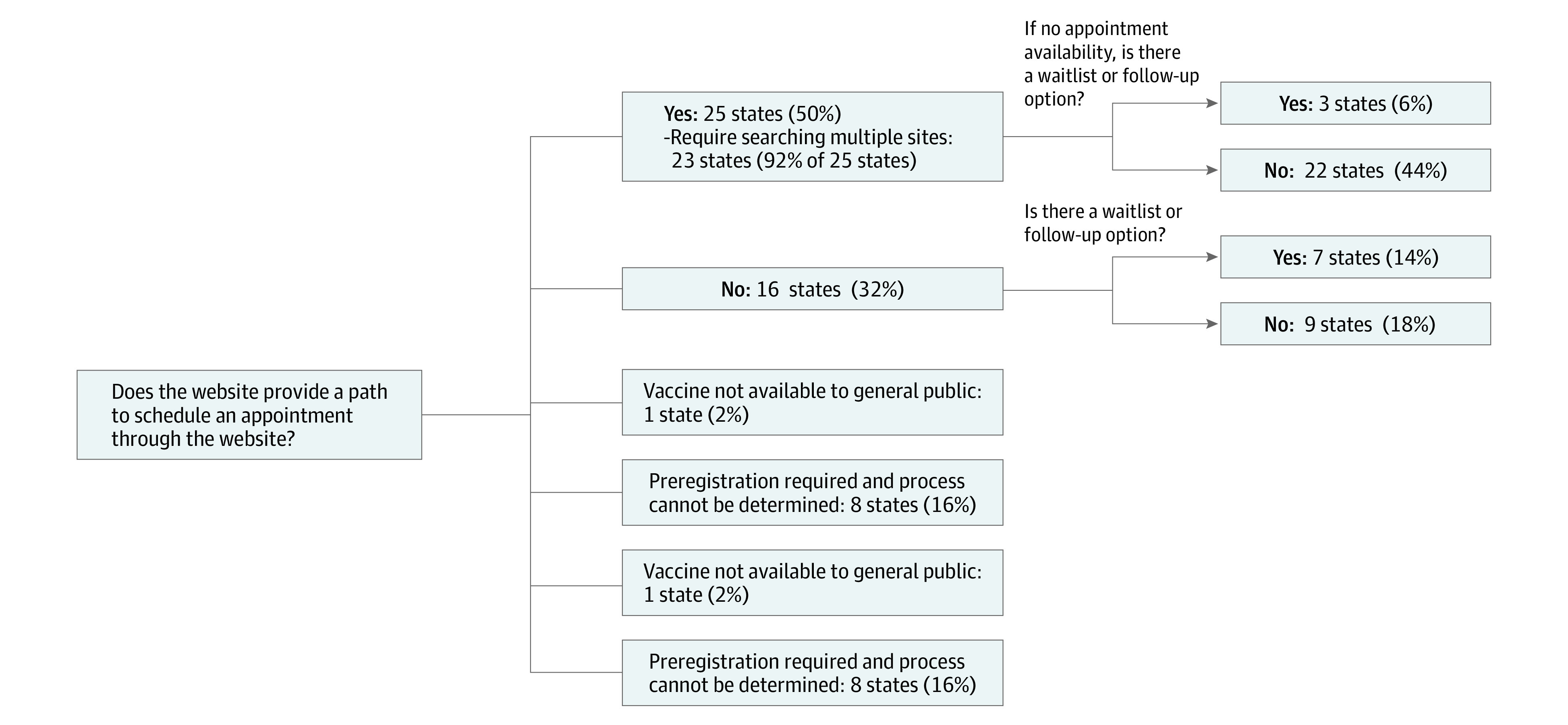
State Websites Providing Web-Based Scheduling and Wait-list of Follow-up Information

Usability evaluation showed that 30 states (60%) had no indicator of when information was last updated. Of the 47 states providing eligibility information, most (27 of 47 [57%]) did not support verifying eligibility. Although 25 states (50%) provided web-based appointment scheduling, the majority (23 of 25 [92%]) required the user to search multiple vaccine locations individually for availability, and only 3 states (6%) had a wait-list or follow-up option when no appointments were available (Figure 2). Nine states (18%) did not offer web-based scheduling or an option for a wait-list or follow-up.

## Discussion

State health department COVID-19 vaccine website accessibility and usability challenges create frustration, may promote health disparities, and contribute to overall ineffective and inequitable distribution. Accessibility issues included a lack of support for smartphone access, English-only text, and poor readability. Usability issues compound the problems by failing to provide critical information, such as the last time or date the site was updated, web-based scheduling, a wait-list or follow-up process, and requiring users to check multiple locations for availability.

To address these issues, websites should support mobile access, offer information in multiple languages, and provide eligibility information in easier to comprehend language. Websites should maintain a last updated line, a clear wait-list or follow-up process, and should not require people to contact numerous vaccine sites for appointments. Adhering to these practices will reduce frustration and improve vaccine distribution. Website features are evolving, and our analysis focused on a specific time window, which is a limitation of the study. Furthermore, people may make appointments through other means.
